# Anticipation may underestimate neuromechanical deficits in chronic ankle instability during walking inversion perturbations

**DOI:** 10.3389/fspor.2026.1852628

**Published:** 2026-07-02

**Authors:** Wenqi Ran, Wei Yin

**Affiliations:** 1 College of Sports and Health, Shandong Sport University, Jinan, China; 2 China National Basketball Academy, Shandong Sport University, Jinan, China

**Keywords:** anticipation, electromyography, kinematics, neuromuscular control, peroneus longus

## Abstract

**Objective:**

This study aimed to compare ankle kinematics and lower limb muscle activation between individuals with and without chronic ankle instability (CAI), both prior to and following an inversion perturbation during walking, with a particular focus on how anticipation modulates muscle activation and the consequent ankle inversion post-perturbation.

**Methods:**

Forty-six participants were enrolled, including 23 with CAI and 23 age- and sex-matched non-CAI controls. They walked at a self-selected pace on a custom-designed platform equipped with a trapdoor programmed to trigger a 24° inversion perturbation under both anticipated and unanticipated conditions. Ankle kinematics and lower limb muscle electromyography were synchronously collected during a 400-ms window, specifically 200 ms pre- and 200 ms post-perturbation. Continuous time-series data were compared using a two-way mixed-design ANOVA via Statistical Parametric Mapping.

**Results:**

Significant interactions were detected in ankle inversion angles (30.0–105.0 ms, F _max_ = 29.981, = 8.337, *p* = 0.005), as well as for the activation of the peroneus longus (PL) (67–121 ms, F _max_ = 25.097, *p* = 0.005) and pre-activation of lateral gastrocnemius (LG) (−200 to −161 ms, F _max_ = 15.694, *p* = 0.014). Transitioning from anticipated to unanticipated conditions elicited an increase in ankle inversion across both individuals with and without CAI, with a significantly greater magnitude of increase observed in CAI individuals (all *p* < 0.001). Individuals without CAI significantly increased their PL activation when transitioning from anticipated to unanticipated conditions (*p* < 0.001), whereas the CAI individuals exhibited no significant change. Conversely, individuals with CAI demonstrated a significant increase in LG pre-activation (*p* = 0.006), while those without CAI exhibited no significant change.

**Conclusion:**

Under unanticipated perturbations, the compensatory increase in LG pre-activation observed in individuals with CAI remains insufficient to offset diminished PL activation and the subsequent increase in ankle inversion. These neuromechanical deficits, typically masked by anticipation, indicate that predictable paradigms may not fully capture real-world injury scenarios. Rehabilitation should prioritize targeted PL recruitment to enhance dynamic stability and reduce reinjury risk.

## 1 Introduction

Lateral ankle sprain (LAS) represents one of the most prevalent musculoskeletal injuries across both athletic and general populations (1). Despite initial recovery, approximately 30% to 70% of individuals develop chronic ankle instability (CAI), a condition characterized by recurrent sprains, frequent “giving way” sensations, and persistent functional limitations (2). Elucidating the neuromechanical mechanisms underlying CAI during dynamic tasks is therefore critical to understanding the pathological basis of reinjury and developing targeted rehabilitation strategies.

Dynamic joint stability relies on highly coordinated neuromuscular control, integrating feedforward pre-activative and feedback reactive responses (3). Individuals with CAI typically exhibit sensorimotor control deficits that directly compromise this dynamic stability (4). Recent evidence suggests these deficits are manifested as simplified or restructured locomotor modules, where altered organizational strategies of muscle synergies hinder the ankle's ability to adapt to dynamic demands (5). Previous research indicates that those with CAI often demonstrate altered neuromuscular control, characterized by increased pre-activation prior to landing (6, 7) or insufficient reactive activation of the lateral ankle muscles following impact (8, 9). These neural deficits may lead to kinematic abnormalities, notably excessive ankle inversion angles, which significantly elevate the risk of recurrent injury during landing (10).

Existing literature investigating the neuromechanical profiles and injury risk of individuals with CAI has predominantly utilized anticipated, pre-planned tasks, such as steady-state walking or predictable landings (11, 12). However, clinical evidence indicates that the majority of actual LASs occur during sudden, unanticipated events (13). This misalignment underscores a notable gap between conventional laboratory assessments and the dynamic, unpredictable environments where real-world reinjury typically occurs. Specifically, a sudden inversion during the heel-contact phase of walking is recognized as a clinically relevant mechanism for these unanticipated sprains (14, 15). Therefore, utilizing unanticipated walking inversion perturbations provides a highly ecologically valid approach for examining how individuals with CAI respond when ankle stability is suddenly challenged.

Neuromuscular characteristics under anticipated and unanticipated conditions may differ, as the efficiency of neuromuscular control strategies is heavily influenced by the ability to predict environmental changes (16). When a perturbation is predictable, the central nervous system (CNS) prioritizes feedforward control by augmenting muscle activation and pre-adjusting joint posture, which potentially masks underlying CAI pathologies through increased joint stiffness (17). Conversely, when predictive cues are absent, dynamic ankle stability depends more on rapid post-perturbation neuromuscular responses (14). Under this condition, deficits in reactive lateral ankle muscle activation may become more apparent and may contribute to excessive ankle inversion (18). Consequently, conclusions from anticipated tasks may not accurately reflect the functional failures driving recurrent sprains in unpredictable, real-world environments (18). Nevertheless, although foundational trapdoor studies have established the relevance of inversion perturbation paradigms and peroneal reflex responses (9, 19), few studies have directly compared anticipated and unanticipated walking inversion perturbations in individuals with CAI. Furthermore, conventional studies often evaluate muscle activation and kinematics in isolation using discrete metrics, which hinders a comprehensive understanding of the dynamic coupling and temporal progression of the interplay between anticipation, neuromuscular control, and mechanical outcomes.

To concurrently bridge these methodological gaps, this study aims to compare the continuous temporal profiles of ankle kinematics and lower leg muscle activation between individuals with and without CAI during walking perturbations, focusing specifically on how anticipation alters these neuromechanical characteristics. A sudden 24° inversion drop was used as a mechanically meaningful perturbation, with its angle selected based on the safe range reported for laboratory ankle inversion simulators and prior evidence that a 20° perturbation can elicit clear peroneal responses (20, 21). We hypothesized that, in the unanticipated condition, individuals with CAI would demonstrate greater inversion angles (Hypothesis #1) and lower reactive activation of the peroneus longus (PL) (Hypothesis #2) during the post-perturbation phase compared to individuals without CAI. In the anticipated condition, individuals with CAI would demonstrate increased lateral muscle pre-activation relative to both controls and their own unanticipated trials (Hypothesis #3).

## 2 Materials and methods

### 2.1 Participants

An *a priori* power analysis was performed for sample size estimation using G*Power software (Version 3.1.9.7, Düsseldorf, Germany). To accurately reflect the specific biomechanical demands of our unexpected walking perturbation paradigm, the required sample size was calculated based on our own pilot study (*n* = 8). Using the primary kinematic outcome (maximum ankle inversion angle), a Group × Condition interaction effect size of Cohen's *f* = 0.345 was derived. By setting a highly rigorous 95% statistical power with an alpha level of 0.05, a minimum requirement of 30 participants was determined to ensure sufficient sensitivity to identify the hypothesized interactions. To ensure superior statistical robustness and account for potential data attrition or individual variability, a larger cohort of 46 participants (23 per group) who met the established inclusion criteria was recruited for this study.

Participants were recruited from a local university through the distribution of flyers and delivery of presentations, yielding 67 people willing to participate in the study. The inclusion criteria for individuals with CAI strictly followed the criteria established by the International Ankle Consortium (22), which included: (a) Aged 18–24 years, inclusive of both sexes; (b) At least two prior lateral ankle sprains, with at least one in the past 12 months that interrupted physical activity for at least one day; (c) At least two episodes of ankle “giving way” within the 6 months preceding enrollment; (d) A CAIT score of less than 24; and (e)Physical activity ≥3 days/week for 90 min within the past 3 months. The inclusion criteria for individuals without CAI were: (a) No history of previous LAS; (b) Matched to the CAI participants in terms of sex, age (±3 years), height (±5 cm), and weight (±5 kg); (c) A CAIT score of 28 or higher; and (d) Physical activity ≥3 days/week for 90 min within the past 3 months. The exclusion criteria for both groups were: (a) A history of lower extremity fracture or surgery; (b) An acute lower extremity injury in the preceding 3 months; (c) Diagnosed neurological, vestibular, or metabolic (e.g., diabetes) disorders; or (d) Bilateral CAI. 46 participants were enrolled, including 23 individuals with CAI (11 females and 12 males, aged 20.3 ± 1.7 years, with a height of 172.1 ± 0.1 cm and a weight of 71.0 ± 13.9 kg; Cumberland Ankle Instability Tool (CAIT) score = 15.0 ± 5.1) and 23 individuals without CAI (12 females and 11 males, aged 21.7 ± 1.8 years, with a height of 171.9 ± 0.1 cm and a weight of 70.7 ± 15.2 kg; CAIT score = 29.0 ± 0.9). There were no significant differences in age, height, or body mass between the groups (*p* > 0.05). All participants provided written informed consent, and the study was approved by the Institutional Review Board of Shandong Sport University (No. 2025053).

### 2.2 Walking trapdoor

**Figure 1:**
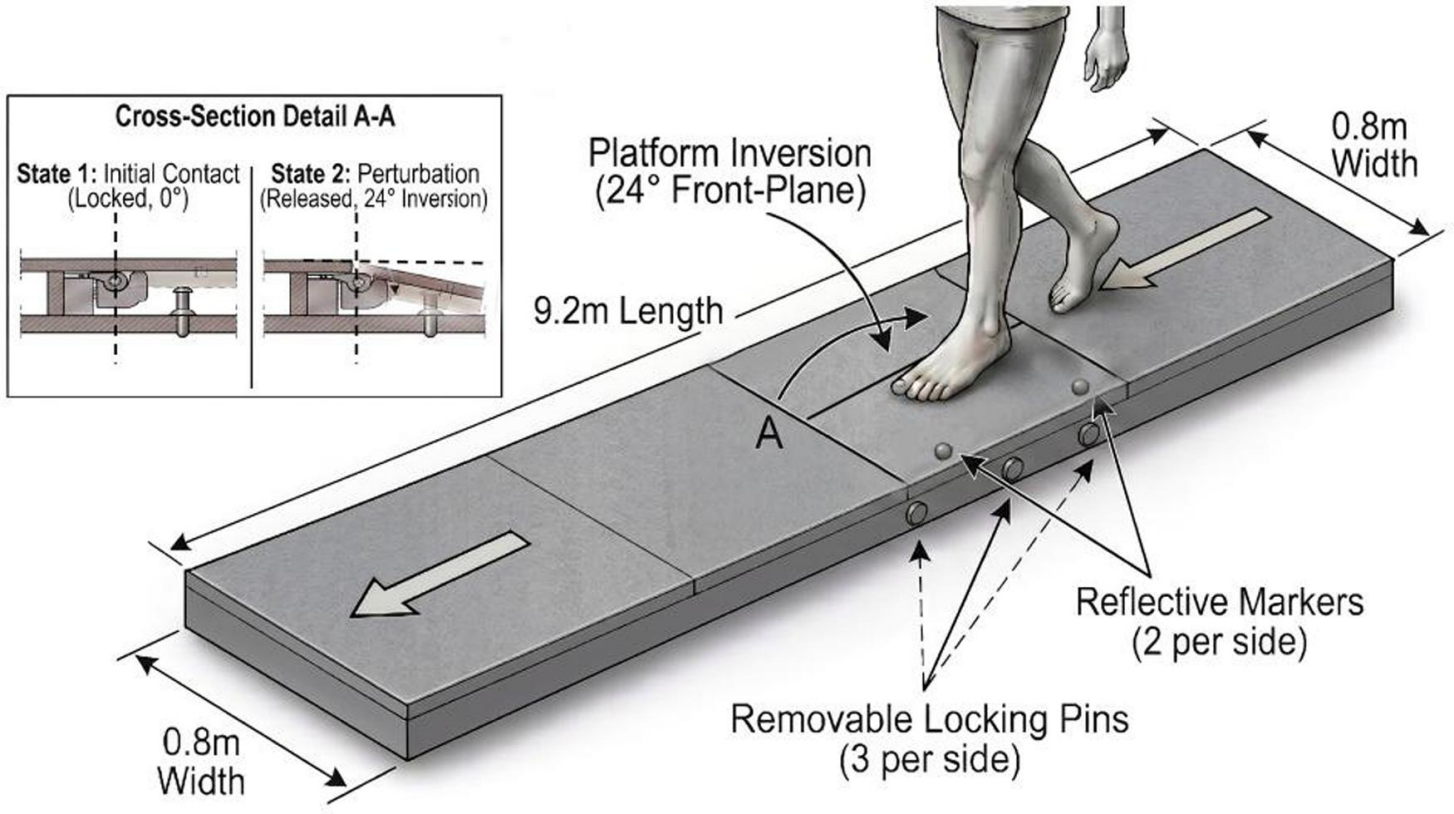
Schematic of the custom-built walkway for ankle inversion perturbations. The walkway (9.2 m length × 0.8 m width) features a trapdoor platform capable of a 24° frontal-plane inversion, triggered by removable locking pins. Reflective markers are integrated for motion capture. The inset provides a cross-sectional view (Detail A-A) of the mechanical structure. Arrows indicate the direction of walking.

A custom walkway measuring 9.2 m length × 0.8 m width was utilized to replicate real-world lateral ankle sprains during walking (Figure 1). The 9.2 m length facilitated several gait cycles, enabling participants to achieve a steady-state walking speed and a natural rhythm prior to the perturbation. An integrated trapdoor mechanism was positioned at the center of the walkway. This tilting system was secured by three removable locking pins at its base; once released, the platform dropped rapidly to induce a 24° inversion in the frontal plane (15). To accurately identify the onset of the perturbation, two reflective markers were attached to the lateral edge of the trapdoor. This specific perturbation angle of 24° was chosen to remain within recognized biomechanical safety limits, especially for participants with CAI, as actual injuries typically occur when ankle inversion exceeds 30° (23).

### 2.3 Protocols

Following informed consent and CAIT questionnaire completion, participants changed into standardized tight-fitting clothing and footwear. Prior to formal testing, familiarization trials were performed to establish a consistent starting position and ensure accurate foot placement on the trapdoor without gait pace alterations. During these practice trials, the perturbation mechanism remained deactivated to minimize anticipatory adaptations. The formal testing protocol comprised unanticipated and anticipated conditions. In the unanticipated condition, participants performed a total of 15 walking trials, including 5 target inversion perturbation trials and 10 “dummy trials” where the trapdoor platform remained locked. While participants were informed that the trial order was randomized, a standardized, blinded sequence was implemented to minimize habituation and overcome anticipation bias, ensuring that at least two dummy trials were presented between each perturbation. To eliminate external environmental cues, participants wore noise-canceling headphones and maintained a fixed gaze on an eye-level target. Immediately following each trial, participants reported their pre-trial expectation; a successful unanticipated event was specifically defined by a predicted stable surface followed by an actual trapdoor drop. If a participant correctly anticipated the perturbation, the trial was discarded and repeated to maintain the validity of the unanticipated state. Conversely, in the anticipated condition, participants were explicitly informed of the upcoming perturbation before each walk. Safety was ensured through a ceiling-mounted harness system and a spotter, and a one-minute rest interval was provided between trials to mitigate the effects of muscle fatigue.

Kinematic data were recorded using a 12-camera Vicon motion capture system (Vicon Motion Systems Ltd., Oxford, UK) sampling at 200 Hz. The assessment was performed on the affected limb of participants with CAI and the dominant limb (defined as the preferred kicking leg) of the controls (24). In strict adherence to the Oxford Foot Model, forty-two retro-reflective markers were placed on specific anatomical landmarks. To ensure consistency and eliminate inter-rater error, all marker placements and anthropometric measurements were conducted by a single experienced researcher.

Surface electromyography (sEMG) signals were captured via a wireless Noraxon system (Noraxon, Scottsdale, AZ, USA) at a sampling rate of 2000Hz. Bipolar electrodes were applied to the muscle bellies of the peroneus longus (PL), lateral gastrocnemius (LG), tibialis anterior (TA), and soleus (SOL), maintaining an inter-electrode spacing of < 2 cm. To optimize signal quality and reduce skin impedance, standard skin preparation procedures, including shaving and cleaning with 70% isopropyl alcohol, were performed prior to electrode attachment.

### 2.4 Data reduction

Data analysis focused on a 400-ms window surrounding the perturbation event, encompassing 200 ms pre- and 200 ms post-perturbation. In this protocol, perturbation onset was defined by the vertical displacement of a reflective marker positioned on the platform's lateral edge. This temporal window captures the critical transition from preparatory feedforward strategies to reactive feedback responses, covering the peak-risk period for LASs (23). To facilitate statistical parametric mapping (SPM) analysis across the entire waveform, all time-series data were time-normalized to a standard length of 401 data points (25).

Kinematic data were smoothed using a 10 Hz low-pass, fourth-order, zero-lag Butterworth filter within the Visual3D environment (v6 Professional, C-Motion, Germantown, MD, USA) (26). Ankle joint kinematics were calculated based on a Cardan rotation order (X-Y-Z), which describes how the distal segment is oriented relative to the proximal segment's reference frame (27).

Raw EMG signals were processed using Noraxon MR3 software (Noraxon, Scottsdale, AZ, USA). The signals were band-pass filtered (10–500 Hz) to minimize noise and smoothed via a 50-ms moving root-mean-square (RMS) window. EMG amplitudes were normalized to the task-specific peak activation recorded from the same muscle across all valid trials within each participant (28). Task-specific peak normalization was selected instead of maximal voluntary isometric contraction (MVIC) normalization because it better aligned with the study's focus on task-evoked reactive activation patterns and avoided potential biases related to AMI susceptibility (29), pain-limited effort (30), and static MVIC reference values during a rapid dynamic perturbation task (31). The processed and normalized waveforms were subsequently exported to MATLAB (R2024b, MathWorks, Natick, MA, USA) for further feature extraction.

### 2.5 Statistics

A two-way mixed-design ANOVA using SPM examined the effects of Group (CAI, Non-CAI) and Condition (Anticipated, Unanticipated) on biomechanical variables. Analyses were performed with open-source SPM1d code (version 0.4, https://www.spm1D.org) in MATLAB (R2024b, MathWorks Inc., USA). Significance thresholds were defined via Random Field Theory (RFT). When significant interactions or main effects emerged, *post-hoc* SPM with Bonferroni's correction addressed multiple comparisons. Independent t-tests compared groups; paired t-tests assessed within-condition differences. Statistical significance was set at *p* < 0.05. Results are reported by listing the temporal clusters where SPM curves exceeded the threshold, including *p*-values for each supra-threshold cluster.

## 3 Results

**Figure 2:**
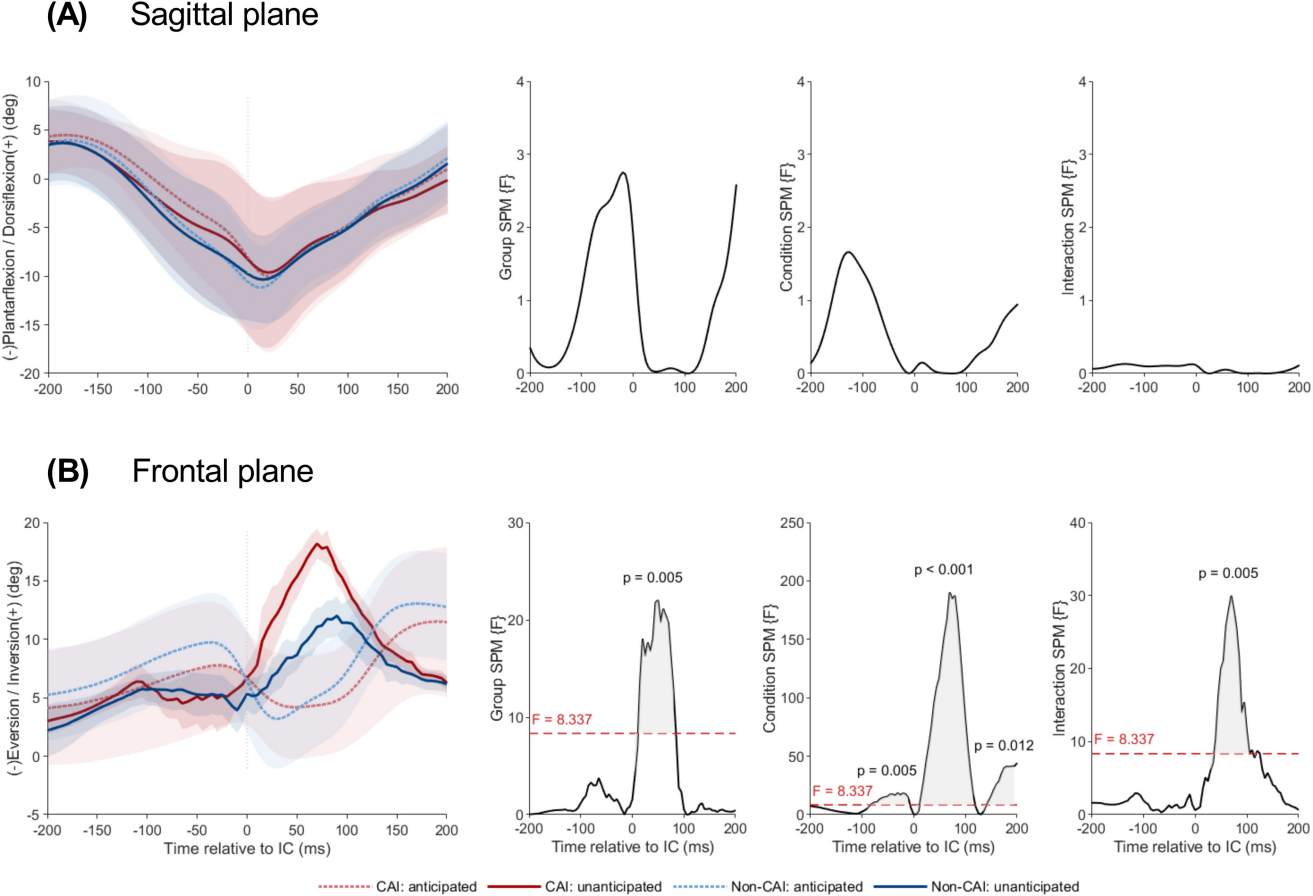
SPM analysis of ankle kinematics in sagittal **(A)** and frontal **(B)** planes. The first column displays the mean and ±1 standard deviation (SD) clouds for the 400-ms window (± 200 ms around initial contact) for CAI and Non-CAI participants across anticipated and unanticipated conditions: CAI anticipated (red dashed), CAI unanticipated (red solid), Non-CAI anticipated (blue dashed), and Non-CAI unanticipated (blue solid). Subsequent columns: SPM {F} results for Group, Condition, and Interaction effects. Red dashed lines denote critical F-thresholds(F crit); gray clusters indicate significant differences (*p* < 0.05).

Analysis of self-selected walking speed revealed no significant differences across groups or conditions (all *p* > 0.05). A significant interaction was detected in ankle inversion angle from 30 to 105 ms (F _max_ = 29.981, *p* = 0.005) during the post-perturbation phase (Figure 2). From anticipated to unanticipated conditions, both individuals with and without CAI increased their ankle inversion angles from 30 to 105 ms, although this increase was greater in individuals with CAI than in those without CAI. A significant group effect was observed from 5 to 80 ms (F _max_ = 22.013, *p* = 0.005) during the post-perturbation phase, with those with CAI having greater inversion angles than those without CAI. A significant condition effect was observed in three intervals, ankle inversion angles were greater in the anticipated condition from −90 to −10 ms (F _max_ = 18.431, *p* = 0.005) and from 135 to 195 ms (F _max_ = 41.541, *p* = 0.012), while the unanticipated condition demonstrated greater inversion from 5 to 115 ms during the post-perturbation phase (F _max_ = 190.146, *p* < 0.001).

**Figure 3:**
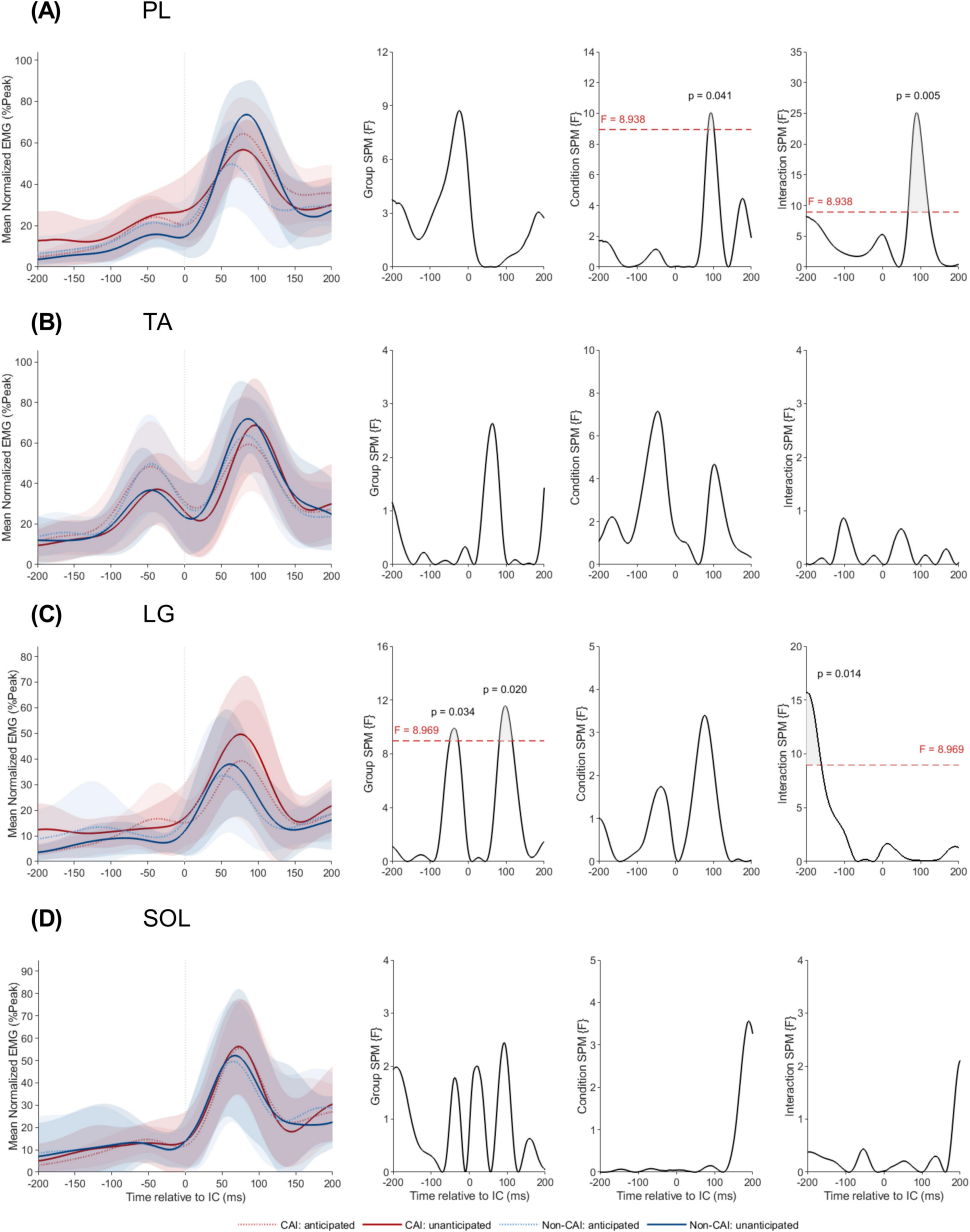
SPM analysis of muscle activation for the **(A)** PL: peroneus longus, **(B)** TA: tibialis anterior, **(C)** LG: lateral gastrocnemius, and **(D)** SOL: soleus. The first column displays the mean and ±1 standard deviation (SD) clouds for the 400-ms window (± 200 ms around initial contact) for CAI and Non-CAI participants across anticipated and unanticipated conditions: CAI anticipated (red dashed), CAI unanticipated (red solid), Non-CAI anticipated (blue dashed), and Non-CAI unanticipated (blue solid). Subsequent columns: SPM {F} results for Group, Condition, and Interaction effects. Red dashed lines denote critical F-thresholds(F crit); gray clusters indicate significant differences (*p* < 0.05).

A significant interaction was detected in PL activation from 67 to 121 ms (F _max_=25.097, *p* = 0.005) during the post-perturbation phase (Figure 3). Individuals without CAI significantly increased their PL activation when transitioning from anticipated to unanticipated conditions (*p* < 0.001), whereas the CAI individuals exhibited no significant change. A significant condition effect was observed from 85 to 101 ms (F _max_=10.044, *p* = 0.041) during the post-perturbation phase, where the unanticipated condition showed higher activation than the anticipated condition (*p* = 0.020).

A significant interaction was detected in LG activation from −200 to −161 ms (F _max_=15.694, *p* = 0.014) during the pre-perturbation phase (Figure 3). From anticipated to unanticipated conditions, individuals with CAI demonstrated a significant increase in LG pre-activation (*p* = 0.006), while those without CAI exhibited no significant change. Significant group effects were detected from −50 to −27 ms (F _max_=9.898, *p* = 0.034) and 81–115 ms (F _max_=11.564, *p* = 0.020), where individuals with CAI exhibited higher LG activation than those without CAI. No significant interaction effects or main effects were observed for the activation of the TA and SOL (*p* > 0.05) (Table 1).

**Table 1:** Statistical details and effect sizes for significant neuromechanical clusters.

Variable	Effect	Phase	Mean _Diff_	F _max_	$\eta_{p}^{2}$	*p*-value
Ankle Kinematics						
Inversion angle	group	5.0 to 80.0 ms	2.94 ± 4.88°	22.013	0.333	0.005
	condition	−90.0 to −10.0	3.05 ± 4.85°	18.431	0.295	0.005
	condition	5.0 to 115.0 ms	−6.55 ± 4.82°	190.146	0.812	<0.001
	condition	135.0 to 195.0 ms	4.27 ± 5.34°	41.541	0.485	0.012
	group*condition	30.0 to 105.0 ms	-	29.981	0.405	0.005
Muscle Activation						
Peroneus Longus	condition	85 ms to 101ms	12.29 ± 32.86%	10.044	0.186	0.041
	group*condition	67 ms to 121ms	-	25.097	0.363	0.005
Lateral Gastrocnemius	group	−50.0 ms to −27ms	6.28 ± 9.48%	9.898	0.184	0.034
	group	81 ms to 115ms	14.92 ± 21.27%	11.564	0.208	0.020
	group*condition	−200 ms to −161 ms	-	15.694	0.263	0.014

Mean _Diff_, mean difference between groups; F _max_, maximum F-statistic within the significant cluster.

## 4 Discussion

To our knowledge, this is the first study to analyze the dynamic kinematics and neuromuscular control characteristics of individuals with CAI during both anticipated and unanticipated walking perturbations. The results supported Hypotheses #1 and #2, confirming that under unanticipated conditions, individuals with CAI exhibited greater inversion and lower reactive activation of the PL during the post-perturbation phase compared to individuals without CAI. Conversely, Hypothesis #3 was rejected because the data revealed that individuals with CAI demonstrated significantly increased preparatory LG activation in the unanticipated condition relative to both the non-CAI group and their own anticipated trials.

Our results demonstrate that anticipation significantly influences ankle kinematics, as evidenced by a marked increase in inversion angles across both individuals with and without CAI when transitioning to unanticipated conditions during the post-perturbation phase. Notably, individuals with CAI exhibited a significantly greater increase in ankle inversion angles than those without CAI. This finding is supported by a previous study (32), which showed that individuals with CAI demonstrated a significantly more inverted ankle orientation exclusively after unplanned landings. Increased ankle inversion observed during unanticipated perturbations may be attributed to the mechanical dysfunction of lateral ankle restraint structures in individuals with CAI (33). Under unanticipated conditions, since the nervous system fails to provide sufficient active protection through immediate muscle contraction, ankle stability becomes highly dependent on passive restraint structures such as ligaments (14, 34). If the ligaments are damaged (especially the anterior talofibular ligament), the abnormal separation between the talus and fibula cannot be limited, leading to joint displacement exceeding normal anatomical limits (35, 36). Furthermore, because functional joint stability depends on the ensemble coding of muscle, cutaneous, and joint afferent inputs, deafferentation in individuals with CAI may delay the immediate perception of joint position changes (37), thereby further exacerbating kinematic deficits. This underlying structural instability is often masked by conscious feedforward strategies during anticipated tasks, yet it is fully manifest in sudden environments where predictive mechanisms are absent (17, 32). The observation that peak inversion angles (approximately 20°) exceeded the 15° injury threshold (38), coupled with an accelerated progression toward this inversion peak in the SPM trajectories (Figure 2), provides a direct biomechanical explanation for the high recurrence rate of LASs (39) in CAI individuals.

This study found that individuals with CAI exhibited greater ankle inversion after an unanticipated perturbation, which may be related not only to inherent mechanical laxity but also to reduced reactive activation of the PL after perturbation. This finding aligns with Palmieri-Smith et al. (9), supporting the view that diminished reactive PL activation is a characteristic neuromuscular feature of ankle instability during sudden inversion challenges. Functionally, rapid resistance to inversion requires the CNS to integrate multisensory afferent inputs from muscle, cutaneous, joint, and ligamentous receptors to support functional joint stability (19, 37). In healthy individuals, this multisensory information can be rapidly organized into a PL-dominant, task-specific stabilizing strategy (40). In contrast, individuals with CAI demonstrated marked attenuation of PL activation within the 67–121 ms window, which corresponds to the medium- and long-latency reflex windows (19, 41). Because these reflex components are mediated by polysynaptic spinal and transcortical long-loop pathways (19), the reduced PL response may indicate impaired reflexive evertor defense. This impairment may be further exacerbated by abnormal angular encoding of cutaneous or joint afferent input from the ankle (42), as well as aberrant afferent signaling from damaged mechanoreceptors that may contribute to arthrogenic muscle inhibition and reduced gamma-loop-mediated spindle sensitivity (29). Together, these sensory and reflex-level deficits may weaken the neural drive required to generate rapid eversion torque, thereby reducing dynamic joint stiffness and allowing greater ankle inversion. Notably, this deficit was more pronounced under unanticipated conditions, which may reflect reduced predictive gating (43). When perturbations are predictable, the CNS may pre-set sensorimotor gain and increase preparatory muscle activation through feedforward regulation (16), partially masking underlying deficits. In the absence of anticipatory cues, however, this compensatory effect is weakened and the underlying reflex impairment becomes more evident (32).

Our EMG results further demonstrated that CAI individuals exhibited significantly higher LG pre-activation compared to those without CAI under unanticipated conditions. This finding can be interpreted within the same integrated sensorimotor framework described above. During sudden inversion perturbations, effective ankle stabilization requires the CNS to integrate multisensory afferent information and organize muscle responses according to the mechanical demands of the task (16), with direction-specific evertor activation being particularly important for resisting inversion (19). In seminal trapdoor studies, healthy individuals exposed to sudden inversion perturbations showed anticipatory and reactive adjustments mainly in muscles directly relevant to ankle stabilization, particularly the tibialis anterior and peroneal muscles, whereas the LG showed little pre-activation and contributed primarily through post-perturbation long-latency reflex responses (40). In contrast, the present CAI group exhibited increased LG pre-activation under unanticipated conditions, despite insufficient reactive PL activation. This pattern suggests that individuals with CAI may fail to organize a task-specific, PL-mediated evertor response and instead rely on a more generalized preparatory stiffening strategy involving the LG (7). Because the LG functions primarily as a plantarflexor rather than a principal evertor (44), its increased recruitment represents a functional mismatch between muscle action and the mechanical demand imposed by sudden inversion (45). Although this strategy may enhance global ankle stiffness through longitudinal joint compression (46), it is likely suboptimal compared with a targeted PL-mediated evertor response and may therefore be insufficient to counteract the substantial inversion torque produced by the perturbation.

Contrary to Hypothesis 3, individuals with CAI did not exhibit higher pre-activation of ankle muscles than individuals without CAI under anticipated conditions, a finding inconsistent with previous perspectives. It is widely held that CAI individuals increase muscle pre-activation to enhance joint stiffness as a means of compensating for mechanical instability (16, 47). This discrepancy may stem from differences in experimental design, as our study randomly interleaved perturbations with normal walking whereas previous studies often utilized single, predictable task patterns (6, 7) that allowed the CNS sufficient time for feedforward regulation. This contextual uncertainty effect may keep CAI individuals in a state of sustained high alert, which potentially reduces CNS sensitivity to anticipatory cues (17). Consequently, rather than executing task-specific adjustments for anticipated trials, the CNS appears to conserve neural resources for more threatening, unanticipated perturbations. This strategy aligns with the increases in LG pre-activation observed under unanticipated conditions in the current study. However, the failure to significantly increase pre-activation of the PL, which is the most critical muscle for resisting inversion, likely stems from inherent muscle inhibition in CAI individuals.

Our results have significant implications for the assessment and clinical practice of CAI. We suggest adopting uncertainty-based paradigms by incorporating random perturbations into dynamic tasks to simulate actual injury-prone scenarios (48). Furthermore, prioritizing the targeted activation of the PL may serve as a critical strategy for enhancing dynamic joint stability and mitigating the risk of reinjury in unpredictable environments.

Several limitations of this study should be acknowledged. First, the absence of “copers” precludes a comprehensive comparison of neuromuscular adaptations across different clinical outcomes of ankle injury. Second, although some residual expectancy may have remained due to post-exposure habituation and the tactile difference between the locked trapdoor and solid ground, our randomized sequence, dummy trials, and expectation checks were used to minimize this influence. Finally, the structural integrity of the calcaneofibular ligament and subtalar joint was not directly assessed. Future studies should combine mechanical laxity assessment with EMG waveform analysis.

## 5 Conclusion

Under unanticipated perturbations, the compensatory increase in LG pre-activation observed in individuals with CAI remains insufficient to offset diminished PL reactive activation and the subsequent increase in ankle inversion. These neuromechanical deficits, typically masked by anticipation, indicate that predictable paradigms may not fully capture real-world injury scenarios. Rehabilitation should prioritize targeted PL reactive recruitment to enhance dynamic stability and reduce the risk of reinjury.

## Data Availability

The raw data supporting the conclusions of this article will be made available by the authors, without undue reservation.
